# Bidirectional association between obstructive sleep apnea and diabetic retinopathy: a severity-stratified systematic review and meta-analysis with GRADE assessment

**DOI:** 10.1007/s00592-026-02732-2

**Published:** 2026-06-23

**Authors:** Abdelaziz Abdelaal, Yahya A. Mahmoud, Weam Alsouri, Mutaz Al-Nawaflh, Kais Al Issa, Luke Yaldo, Osama Elaraby, Hashem Abu Serhan

**Affiliations:** 1 Harvard Medical School, Boston, MA USA; 2 Faculty of Medicine, Tanta University, Tanta, El-Gharbia Egypt; 3 Tanta Research Team, Tanta, El-Gharbia Egypt; 4 Faculty of Medicine, Al-Azher University, Gaza, Palestine; 5 Al Shifa Hospital, Gaza, Palestine; 6 Department of Ophthalmology, King Hussein Medical Center, Amman, Jordan; 7 Faculty of Medicine, Aleppo University, Aleppo, Syria; 8 Bascom Palmer Eye Institute, University of Miami Miller School of Medicine, Miami, FL USA; 9 Department of Ophthalmology, Byers Eye Institute, Stanford University School of Medicine, Palo Alto, CA USA; 10 Department of Ophthalmology, Hamad Medical Corporation, Hamad, Qatar

**Keywords:** Obstructive sleep apnea, Diabetic retinopathy, Polysomnography, Retina, Diabetes mellites

## Abstract

**Purpose:**

To synthesise the bidirectional evidence on the association between obstructive sleep apnea (OSA) and diabetic retinopathy (DR), separating studies framing OSA as the exposure for DR (Arm 1) from studies framing DR as the exposure for OSA (Arm 2).

**Methods:**

Systematic review and meta-analysis of observational studies (PROSPERO: CRD420251135427). PubMed, Scopus, Web of Science, and Google Scholar were searched from inception to July 13, 2025, supplemented by structured re-screening of five prior systematic reviews and forward and backward citation searching. Eligible studies enrolled individuals with diabetes mellitus and reported binary OSA (or, in Arm 2, DR) exposure with an unexposed comparator. Within each arm, exposure and outcome were severity-stratified; unadjusted and adjusted odds ratios (ORs) were pooled separately using random-effects models in MetaAnalysisOnline.com. Risk of bias was assessed with the Newcastle–Ottawa Scale (NOS) and certainty of evidence with the Grading of Recommendations Assessment, Development, and Evaluation (GRADE) framework.

**Results:**

Twenty-six studies met eligibility criteria. In Arm 1 (OSA → DR), the unadjusted pooled OR for any DR with OSA classified as a binary exposure was 2.41 (95% confidence interval [CI] 1.53–3.80; 10 studies; very low certainty), but attenuated to 1.03 (95% CI 0.64–1.66; 2 studies; low certainty) in confounder-adjusted analyses. OSA-severity-stratified Arm 1 estimates for mild, moderate, and severe OSA were directionally consistent but not statistically significant in either unadjusted or adjusted pooling. DR-stage-specific Arm 1 analyses (background DR, mild/moderate/severe non-proliferative DR [NPDR], proliferative DR [PDR]) were directionally aligned with the overall Arm 1 finding but imprecise. In Arm 2 (DR → OSA), pooled estimates showed inverse or null associations across DR severity strata; severe OSA as an outcome of DR yielded a pooled OR of 1.24 (95% CI 0.92–1.68; 4 studies; low certainty).

**Conclusions:**

The OSA–DR association is sensitive to the direction of the framed exposure–outcome relationship, to confounder adjustment, and to severity classification. Substantial attenuation of the unadjusted Arm 1 signal after adjustment, together with the inverse or null Arm 2 pattern, indicates that previously reported pooled estimates were influenced by confounding and by directional misclassification. Polysomnography-confirmed prospective cohorts with uniform DR grading and individual-participant-level adjustment for body mass index, diabetes duration, and metabolic comorbidities are needed to clarify the direction and magnitude of the relationship.

**Supplementary Information:**

The online version contains supplementary material available at https://doi.org/10.1007/s00592-026-02732-2.

## Introduction

Diabetic retinopathy (DR) remains a leading cause of preventable blindness worldwide, affecting an estimated 103 million adults in 2020 and projected to reach ~ 161 million by 2045 [1], underscoring the need to clarify modifiable risks beyond glycemic control. The development and progression of DR have been associated with numerous host risk factors including Obstructive sleep apnea (OSA) [2]. OSA is a chronic sleep-related breathing disorder characterised by recurrent upper-airway collapse during sleep that induces intermittent hypoxaemia and sleep fragmentation [3], resulting in sympathetic activation, oxidative stress, inflammation, and endothelial dysfunction, with consequent upregulation of hypoxia-inducible factor–1α, vascular endothelial growth factor, and other pro-angiogenic mediators [4, 5]. OSA is more prevalent in patients with diabetes (58%–86%) than in the general population and has been implicated as an independent contributor to insulin resistance and poor glycaemic control [6, 7]. It has additionally been associated with other diabetes-related microvascular complications, supporting biological plausibility for an OSA–DR link [8].

Epidemiologic evidence relating OSA to DR has been inconsistent. An earlier meta-analysis reported approximately a two-fold higher odds of DR among patients with OSA, based on a small number of studies [9]. Subsequent syntheses and narrative reviews emphasised heterogeneity across designs, OSA and DR ascertainment methods, and adjustment strategies, concluding that the link — while biologically credible — remained methodologically weak and at times contradictory [1, 9–11]. Emerging clinical observations further suggest that hypoxemia metrics (e.g., minimum nocturnal SpO₂ or rapid eye movement-apnea hypopnea index (REM-AHI)) may relate more strongly to advanced retinopathy than AHI alone, and that effective OSA therapy could modify vascular dysfunction, but definitive data are limited [5].

Several knowledge gaps persist that the present synthesis was designed to address. First, prior meta-analyses pooled studies framing OSA as the exposure for DR together with studies framing DR as the exposure for OSA, despite the fundamentally different epidemiological questions these designs ask. Second, prior reviews commonly pooled crude and confounder-adjusted estimates within a single forest plot, obscuring the role of confounding in the observed association [4, 5, 9]. Third, severity stratification — both for the OSA exposure (mild/moderate/severe by AHI thresholds) and for the DR outcome (background, mild/moderate/severe non-proliferative DR [NPDR], proliferative DR [PDR]) — has been incomplete or absent. Fourth, formal certainty-of-evidence rating at the per-stratum level has rarely been performed.

We therefore conducted a systematic review and meta-analysis with five methodological refinements relative to prior work: (i) bidirectional analytical structure, separating studies framing OSA as the exposure for DR (Arm 1: OSA → DR) from studies framing DR as the exposure for OSA (Arm 2: DR → OSA); (ii) dual severity stratification of both exposure and outcome within each arm; (iii) separate pooling of unadjusted and confounder-adjusted estimates to make the contribution of confounding visible to readers; (iv) GRADE certainty rating at the stratum level rather than for the synthesis as a whole; and (v) expansion of the evidence base through structured re-screening of five prior systematic reviews and an updated search to July 2025. Together, these refinements were intended to move beyond a single pooled “any OSA → any DR” estimate toward a structured map of where the evidence is strongest, where it is inconsistent, and where directionality complicates clinical interpretation.

## Methods

### Study design and reporting

We conducted a systematic review and meta-analysis of observational studies examining the association between OSA and DR. Methods followed PRISMA 2020 guidance [12]; a protocol and analysis plan were specified a priori. The study protocol was registered on PROSPERO (CRD420251135427). As the study synthesized published aggregate data, ethics approval was not required.

## Data sources and search strategy

We searched PubMed, Scopus, Web of Science, and Google Scholar from database inception to July 13, 2025 with no language, geography, age, or publication-status restrictions. The PubMed strategy combined controlled vocabulary and text words for OSA (e.g., “sleep apnea/apnoea,” “obstructive sleep,” OSAHS, hypopnea/hypopnoea; MeSH terms Sleep Apnea, Obstructive and Sleep Apnea Syndromes) AND DR (text words and MeSH Diabetic Retinopathy). Platform-specific adaptations were used for Scopus and Web of Science (Boolean ALL/TS fields). To maximize sensitivity during screening, we also searched for broader or “remote” terms that can conceal DR data in full texts or supplements, including “diseases of the retina,” “systemic/retinal complications,” “retinal diabetopathy,” “diabetic complications,” “microangiopathy,” “ocular manifestations,” and “retinal microvascular signs/complications.” Because DR data are frequently reported only within tables or appendices, we screened full texts and all supplementary materials when titles/abstracts were ambiguous. The complete search query can be found in E-supplement 1. References of eligible reports and relevant reviews were hand-searched for additional studies [13, 14].

## Eligibility criteria

The inclusion criteria were set according to the PICOS (population, exposure, comparison, outcomes, and study design) framework [15].

- Population: Individuals with diabetes mellitus (type 1 or type 2) in any care setting.
- Exposure (Arm 1): OSA as defined by the original study, including laboratory polysomnography (gold standard), home sleep testing, validated questionnaires or clinical diagnoses, or administrative/ICD coding. When available, OSA severity was harmonised to AHI thresholds: mild (5 to < 15 events/h), moderate (15 to < 30), severe (≥ 30), and “moderate-to-severe” (> 15). For Arm 2, the exposure was DR (with severity stratified to background DR, mild/moderate/severe NPDR, PDR, or DR not classified by severity, as reported by original authors).
- Comparator: Participants without the relevant exposure (non-OSA controls in Arm 1; no-DR controls in Arm 2).
- Outcomes (Arm 1): Any DR (presence vs. absence) and DR stage (no DR/D0; mild NPDR/D1; moderate NPDR/D2; severe NPDR/D3; PDR/D4). Outcome (Arm 2): OSA presence and OSA severity (mild/moderate/severe). Diabetic macular edema (DME) was not considered. Outcomes were accepted as defined by authors; where specified, they aligned with International Clinical Diabetic Retinopathy (ICDR) or Early Treatment Diabetic Retinopathy Study (ETDRS) criteria [16].
- Study designs: Observational analytic studies (cross-sectional, cohort, and case–control).

Studies were excluded if they: reported OSA only as a continuous AHI variable without binary classification against an unexposed comparator; reported DR data only as a composite of microvascular complications; focused exclusively on DME without separately extractable DR data; comprised an OSA-only or DR-only cohort with no unexposed comparator group; or used a comparator other than “no OSA”/“no DR” (for example, high-risk versus low-risk OSA without an unexposed group). Case reports and case series, editorials, narrative reviews, and non-human studies were also excluded. When multiple publications overlapped in population and timeframe, the most comprehensive dataset was retained.

## Bidirectional analytical structure

A central methodological refinement of this synthesis was the explicit separation of studies into two analytical arms based on how the original authors framed and analysed the exposure–outcome relationship. Arm 1 (OSA → DR) comprised studies in which OSA (or OSA severity) was the regressor and DR (or DR stage) was the regressand. Arm 2 (DR → OSA) comprised studies in which DR (or DR severity) was the regressor and OSA (or OSA severity) was the regressand. Allocation to an arm was determined by the original authors’ analytical framing rather than by re-modelling cross-sectional data into the opposite causal direction.

## Study selection

Titles/abstracts and full texts were screened independently in duplicate (Y.A.M., W.A.), with disagreements resolved by consensus or a third reviewer (A.A.). Full texts and supplements were reviewed whenever titles/abstracts were non-specific yet potentially relevant under the broadened term set noted above. Reasons for exclusion at full-text review were recorded.

### Data extraction

Two reviewers independently extracted (Y.A.M., W.A.), using a piloted form: study characteristics (country, year of investigation, design, setting); population details (sample size, age inclusion criterion, mean age, sex, diabetes type and duration, glycated haemoglobin [HbA1c], body mass index (BMI); exposure ascertainment (polysomnography vs. alternative); OSA severity categories (AHI thresholds); outcome definitions (ICDR/ETDRS or author-defined); confounder-adjustment set; matching strategy (including propensity-score matching [PSM]); and outcome data. Body mass index was a prespecified key confounder for adjusted analyses. For effect sizes, odds ratios (ORs) with 95% confidence intervals (CIs) were extracted or derived. When a primary study reported a 2 × 2 contingency table rather than a crude OR, the table was converted to OR with 95% CI in R prior to entry into the meta-analysis, ensuring that all crude estimates contributing to a pooled stratum were computed using the same algorithm (E-supplements 3–4) [17]. When only adjusted estimates were reported, they were entered using a generic inverse-variance approach with the covariate set recorded. For Chen 2020, the relevant numerical estimate was reported only graphically; a senior reviewer (A.A.) independently extracted values using PlotDigitizer (https://plotdigitizer.com/) [18]. All extractions were cross-checked, and discrepancies were resolved by consensus against the source publication.

## Risk of bias appraisal

Two reviewers independently assessed risk of bias using the Newcastle–Ottawa Scale (NOS) [19], applying the cohort or case-control version as appropriate. NOS domain scores were translated to Agency for Healthcare Research and Quality (AHRQ) categories using prespecified thresholds: good quality required 3–4 stars in the selection domain, 1–2 stars in comparability, and 2–3 stars in outcome/exposure; fair quality required 2 stars in selection, 1–2 stars in comparability, and 2–3 stars in outcome/exposure; poor quality was assigned for 0–1 star in selection, or 0 stars in comparability, or 0–1 star in outcome/exposure [20]. Quality judgements informed sensitivity analyses and contributed to certainty ratings.

## Outcomes and effect measures

The primary effect measure in each arm was the OR for the relevant exposure–outcome pairing. Within Arm 1, analyses were stratified by OSA severity (mild, moderate, severe, not classified) for the primary “any DR” outcome, and by DR stage (background DR, mild/moderate/severe NPDR, overall NPDR, PDR) for stage-specific outcomes. Within Arm 2, analyses were stratified by OSA severity outcome (mild, moderate, severe) for the primary DR-as-exposure pairing, and by DR severity exposure (mild/moderate/severe NPDR, PDR, DR not classified) for the overall OSA outcome. Within each stratum, unadjusted and confounder-adjusted estimates were pooled separately.

### Statistical analysis

Pooled analyses used random-effects models with the DerSimonian–Laird estimator for between-study variance (τ²), generating summary ORs with 95% CIs. Heterogeneity was quantified using I². Strata containing only a single study were reported narratively rather than as pooled estimates; this convention applied to several DR-severity strata in Arm 2 in which only one study contributed an estimate. Analyses were performed using MetaAnalysisOnline.com [17], a web-based platform implementing standard meta-analytic procedures (DerSimonian–Laird, Mantel–Haenszel, and inverse-variance methods) for binary outcome data.

We did not perform multivariable meta-regression to evaluate diabetes type, BMI, age, or duration of diabetes as moderators. Methodological guidance recommends a minimum of approximately 10 studies per covariate for reliable meta-regression [21]; most stratified analyses in this synthesis contained 2–9 studies, and aggregate-level meta-regression on individual-level confounders such as BMI is additionally subject to ecological fallacy. We instead used prespecified subgroup pooling and reported the heterogeneity of population characteristics narratively in the Results.

Small-study effects were not formally evaluated using funnel-plot asymmetry or Egger’s test for two reasons that are now stated explicitly. First, most stratified analyses contained fewer than 10 studies, below the threshold at which these tests are reliably interpretable. Second, in the strata that did contain ≥ 10 studies, individual studies contributed multiple effect estimates across severity and adjustment subgroups, violating the independence assumption underlying funnel-plot tests. All tests were two-sided with α = 0.05.

### Certainty of evidence

Certainty of evidence was assessed using the Grading of Recommendations Assessment, Development, and Evaluation (GRADE) framework [22] at the stratum level rather than for the synthesis as a whole. Observational evidence was started at “low” and rated down for risk of bias (residual confounding, NOS quality), inconsistency (I² and overlap of CIs), indirectness (population and definition heterogeneity), imprecision (95% CI width and optimal information size), and suspected publication bias. Rating up was considered for large effects or evidence of a consistent severity gradient. Final certainty ratings (very low/low/moderate/high) are reported alongside each pooled estimate in Tables 1 and 2 and in the Results.

**Table 1:** Pooled effect estimates and GRADE certainty assessments for Arm 1 of the bidirectional meta-analysis: studies framing obstructive sleep apnea (OSA) as the exposure for diabetic retinopathy (DR)

Outcome	Studies	OR (95% CI)	I^2^ (%)	Figure	Certainty (GRAE)
Overall DR - Unadjusted risk				Figure 2A	
Exposure: OSA (mild)	2	1.56 (0.90, 2.72)	0%		Low
Exposure: OSA (moderate)	2	1.29 (0.52, 3.21)	47.60%		Very low
Exposure: OSA (severe)	4	1.51 (0.94, 2.42)	0%		Low
Exposure: OSA (not classified)	10	2.41 (1.53, 3.80)	88.80%		Very low
Overall DR - Adjusted risk				Figure 2B	
Exposure: OSA (mild)	2	1.28 (0.68, 2.41)	0%		Low
Exposure: OSA (moderate)	2	1.12 (0.31, 3.98)	66.60%		Very low
Exposure: OSA (severe)	3	1.30 (0.68, 2.45)	0%		Low
Exposure: OSA (not classified)	2	1.03 (0.64, 1.66)	0%		Low
Background DR - Unadjusted risk				Figure 3A	
Exposure: OSA (not classified)	2	0.59 (0.35, 1.00)	0%		Low
Overall NPDR - Unadjusted risk				Figure 3E	
Exposure: OSA (not classified)	4	2.93 (0.88, 9.74)	56%		Very low
Mild NPDR - Unadjusted risk				Figure 3B	
Exposure: OSA (not classified)	2	1.02 (0.51, 2.03)	0%		Low
Moderate NPDR - Unadjusted risk				Figure 3C	
Exposure: OSA (not classified)	2	0.70 (0.33, 1.51)	0%		Low
Severe NPDR - Unadjusted risk				Figure 3D	
Exposure: OSA (not classified)	2	2.39 (0.74, 7.73)	0%		Low
Overall NPDR - Unadjusted risk				Figure 3E	
Exposure: OSA (not classified)	4	2.93 (0.88, 9.74)	56%		Very low
PDR - Unadjusted risk				E-supplement 5	
Exposure: OSA (not classified)	9	1.83 (0.79, 4.26)	88%		Very low
PDR - Adjusted risk				E-supplement 6	
Exposure: OSA (not classified)	2	0.96 (0.19, 4.96)	94.60%		Very low

Estimates are presented for any DR stratified by OSA severity (mild, moderate, severe, not classified) with unadjusted and confounder-adjusted analyses pooled separately, and for stage-specific DR outcomes (background DR, mild/moderate/severe non-proliferative DR [NPDR], overall NPDR, and proliferative DR [PDR]) with OSA classified as a binary exposure

*OSA* obstructive sleep apnea, *DR* diabetic retinopathy, *NPDR* non-proliferative (pre-proliferative) DR, *PDR* proliferative DR, *OR* odds ratio, *CI* confidence interval, *GRADE* Grading of Recommendations Assessment, Development, and Evaluation

**Table 2:** Pooled effect estimates and GRADE certainty assessments for Arm 2 of the bidirectional meta-analysis: studies framing diabetic retinopathy (DR) as the exposure for obstructive sleep apnea (OSA)

Outcome	Studies	OR (95% CI)	I^2^ (%)	Figure	Certainty (GRAE)
Mild OSA				E-supplement 7	
Exposure: DR (not classified)	2	0.66 (0.35, 1.26)	70.40%		Very low
Moderate OSA				E-supplement 8	
Exposure: DR (not classified)	3	0.99 (0.49, 2.00)	70.20%		Very Low
Severe OSA				E-supplement 9	
Exposure: DR (not classified)	4	1.24 (0.92, 1.68)	0%		Low
Overall OSA - Unadjusted risk				Figure 4	
Exposure: NPDR (mild)	1	0.70 (0.63, 0.77)	N/A		Very low
Exposure: NPDR (moderate)	4	0.77 (0.39, 1.54)	51.40%		Very low
Exposure: NPDR (severe)	1	0.67 (0.41, 1.09)	N/A		Very low
Exposure: PDR	3	0.71 (0.39, 1.29)	85.10%		Very low
Exposure: DR (not classified)	2	0.64 (0.59, 0.70)	0%		Low
Overall OSA - Adjusted risk				Figure 5	
Exposure: NPDR (mild)	1	0.64 (0.58, 0.71)	N/A		Very low
Exposure: NPDR (moderate)	2	0.72 (0.21, 2.41)	82.20%		Very low
Exposure: NPDR (severe)	1	0.48 (0.29, 0.78)	N/A		Very low
Exposure: PDR	3	0.87 (0.46, 1.67)	80.30%		Very low
Exposure: DR (not classified)	1	0.57 (0.52, 0.62)	N/A		Very low

Estimates are presented for OSA severity outcomes (mild, moderate, severe) with DR classified as a binary exposure, and for overall OSA stratified by DR severity exposure (mild/moderate/severe non-proliferative DR [NPDR], proliferative DR [PDR], DR not classified) with unadjusted and confounder-adjusted analyses pooled separately. Strata containing a single contributing study are indicated and reported narratively in the Results rather than as pooled meta-analytic estimates

*OSA* obstructive sleep apnea, *DR* diabetic retinopathy, *NPDR* non-proliferative (pre-proliferative) DR, *PDR* proliferative DR, *OR* odds ratio, *CI* confidence interval, *GRADE* Grading of Recommendations Assessment, Development, and Evaluation

## Results

### Summary of key findings

Twenty-six observational studies including 706,105 individuals with diabetes were synthesised in a bidirectional meta-analysis (Tables 1 and 2). In studies framing OSA as the exposure for DR (Arm 1), the unadjusted pooled odds of any DR were higher among patients with OSA (OR 2.41, 95% CI 1.53–3.80; 10 studies), but this signal attenuated substantially in confounder-adjusted analyses (OR 1.03, 95% CI 0.64–1.66; 2 studies). When OSA was stratified by severity, mild, moderate, and severe categories yielded directionally similar but statistically non-significant estimates in both unadjusted and adjusted analyses. DR stage-specific estimates within Arm 1 were directionally aligned with the overall finding but imprecise. In studies framing DR as the exposure for OSA (Arm 2), pooled estimates were inverse or null across DR severity strata; severe OSA as an outcome of DR yielded a non-significant pooled OR of 1.24 (95% CI 0.92–1.68). Overall, the OSA–DR association was sensitive to the direction of the framed exposure–outcome relationship and to confounder adjustment, with certainty of evidence rated low to very low across most strata.

### Literature search results

**Fig. 1:**
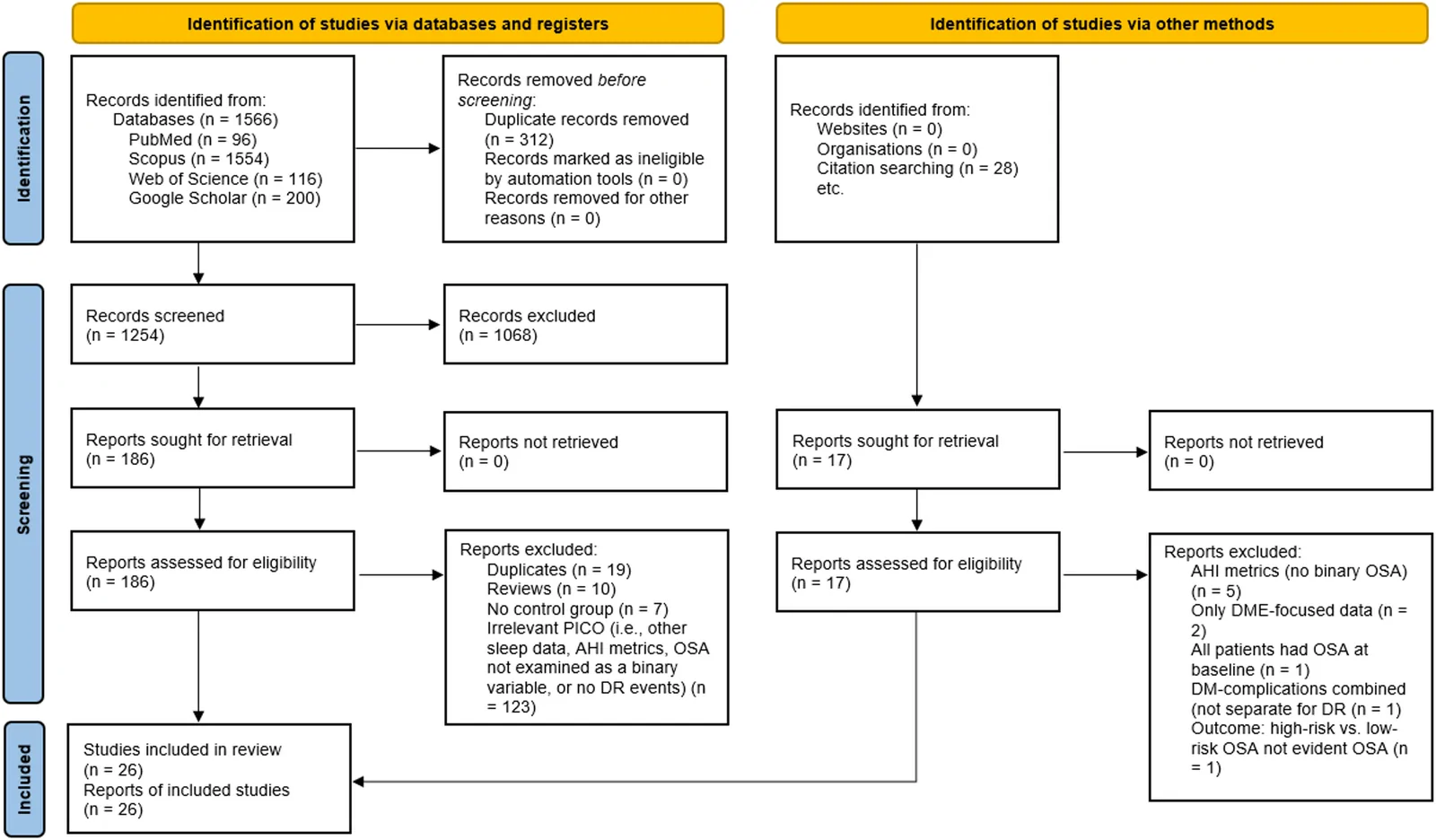
PRISMA 2020 flow diagram of the systematic search and study selection process. Database searching of PubMed, Scopus, Web of Science, and Google Scholar yielded 1,566 records; an additional 17 records were identified through citation searching of five prior systematic reviews and the reference lists of all eligible reports. After duplicate removal and screening against the prespecified PICOS eligibility criteria, 26 studies were included in the bidirectional meta-analysis

The database search yielded 1,566 citations, of which 312 duplicates were removed, leaving 1,254 records for screening (Fig. 1). Of the 186 full texts retrieved from database searching, 167 were excluded (123 for irrelevant population, exposure, or outcome, including studies reporting OSA only as a continuous AHI variable, OSA-only or DR-only cohorts, or DR data not separately extractable from composite microvascular outcomes; 19 duplicates; 10 reviews; 7 with no control group), leaving 19 eligible studies from this stream. Citation searching of five prior systematic reviews and the reference lists of all eligible reports identified 17 additional candidate reports, of which 10 were excluded (5 for reporting AHI metrics without binary OSA classification; 2 for DME-focused outcomes; 2 for OSA-only cohorts without an unexposed comparator; and 1 for a comparator other than “no OSA”). Seven studies from the citation-search stream met all eligibility criteria and were included. In total, 26 studies contributed to the bidirectional synthesis [23–48].

### Baseline characteristics

Baseline characteristics of the included studies are summarised in Table 3. Most reports were retrospective cohort or cross-sectional studies, with three prospective cohorts [24, 38, 42] and one case-control study [34]. Studies spanned North America, Europe, Asia, and North Africa, including the United States, Canada, the United Kingdom, France, Belgium, Denmark, China, India, Japan, Singapore, and Tunisia. Years of investigation ranged from the early 2000 s to 2020.

**Table 3:** Baseline characteristics of the 26 studies included in the bidirectional meta-analysis of Obstructive Sleep Apnea (OSA) and Diabetic Retinopathy (DR)

Study	Exposure	Country	YOI	Design	Population	Sample size			Age; m (SD)		Male (%)	
						Total	Exposure	Control	Exposure	Control	Exposure	Control
Chew 2019	DR	Singapore	-	CS	T1DM/T2DM, ≥ 21 yrs	92	29	63	56.9 (7.7)	59.0 (9.4)	-	-
Grauslund 2021	DR	Denmark	2013–2018	CS	T2DM	153,238	13,538	139,700	62.57 (10.53)	66.50 (11.79)	55.8	-
Nishimura 2015	DR	Japan	2010–2012	CS	T2DM, 20–80 yrs	136	37	99	53.9 (12.3)	58.1 (12.4)	84.8	81.1
Chang 2018	DR	USA	2008–2016	RC	T1DM/T2DM, > 18 yrs	317	220	97	-	-	-	37.1
Baba 2015	OSA	Tunisia	-	RC	T2DM	60	20	40	59 (-)	59 (-)	-	-
Ba-Ali 2023	OSA	Denmark	1980–2014	RC	T1DM/T2DM	532,828	13,279	519,549	55.6 (12.5)	61.6 (17.6)	75.6	51.9
Du 2020	OSA	China	March-2017	CS	T2DM	330	217	113	-	-		
El Ouardighi 2022	OSA	Belgium	2017–2020	CC	T2DM, ≥ 18 yrs	60	30	30	61.67 (8.56)	62.10 (10.28)	50	50
Kaswan 2021	OSA	India	2019–2020	CS	T1DM/T2DM, > 18 yrs	362	18	344	55.83 (9.7)	55.84 (9.9)	83.3	63.7
Zhang et 2014	OSA	China	2010–2011	RC	T2DM, 20–80 yrs	472	310	162	56.2 (10.5)	51.4 (12.2)	69.4	65.4
Yang 2022	OSA	Canada	2018–2020	CS	DM2 patients	32	17	15	62 (9.3)	60 (7.8)	94	47
Xue 2020	OSA	China	2010–2018	CS	T2DM, 18–85 yrs	463	455	8	55.6 (14.0)	62.7 (11.4)	63.2	61.8
Pépin 2023	OSA	France	2016–2020	PC	T1DM, > 18 yrs	769	121	648	Mild to moderate: 61.33 (14.30) Severe; 63.67 (12.55)	46.67 (17.09)	54.5	44.3
Rudrappa 2015	OSA	UK	-	CS	T2DM obese	31	17	16	54.1 (-)	55.8 (-)	100	100
West 2010	OSA	UK	2004–2005	RC	T2DM, 18–75 yrs	118	28	90	67.2 (8.8)	66.3 (10.2)	100	100
Manin 2014	OSA	France	2011–2012	CS	T1DM, > 30 yrs	67	31	36	56 (10)	53 (10)	68	53
Banerjee 2013	OSA	UK	2009–2011	RC	T2DM	93	47	46	54 (10)	49 (10)	56.5	25
Blegen 2021	OSA	USA	2015–2018	CS	T1DM/T2DM	150	32	118	64.0 (10.2)	65.7 (11.2)	68.8	47.5
Chen 2020	OSA	China	2017–2019	RC	DFU	127	-	-	-	-	-	-
Banghoej 2016	OSA	Denmark	2013–2014	RC	T1DM, > 18 yrs	199	92	107	59 (10)	46 (15)	76	60
Altaf 2017 2017	OSA	UK	2014	PC	T2DM	230	147	83	58.7 (11.4)	54.7 (11.9)	66	41
Furukawa 2013	OSA	Japan	2009–2010	RC	T2DM, 20–85 yrs	513	233	280	3.4 (10)	60.9 (10.8)	57.3	56.7
Hermans 2014	OSA	Belgium	-	CS	T2DM, adults	815	129	686	64	67.5 (-)	77.5	64.3
Storgaard 2014	OSA	Denmark	2010–2011	CS	T2DM	183	106	77	58.3 (9.6)	60.4 (11.8)	63	55
Laaban 2009	OSA	France	2007	RC	T2DM	303	160	112	-	60 (12)	58.1	40
Adderley 2020	OSA	UK	2005–2018	RC	T2DM ≥ 16 yrs	14,117	3667	10,450	60.07 (10.56)	60.99 (9.08)	73.06	75.73

Studies are listed by analytical arm (DR-as-exposure or OSA-as-exposure), with country, year of investigation, study design, study population (including diabetes type and age inclusion criterion), sample size for exposure and control groups, mean age, and proportion of male participants

*YOP* year of publication, *YOI* year of investigation, *RC* retrospective cohort, *OSA* obstructive sleep apnea, *CC* case-control, *PC* prospective cohort, *CS* cross-sectional, *m (SD)* mean (standard deviation), *UK* United Kingdom, *USA* United States of America, *DM* diabetes mellitus, *DFU* diabetic foot ulcer, *yr* year, *DR* diabetic retinopathy

In total, 706,105 individuals were included across the 26 studies. Of these, 17 studies enrolled exclusively patients with type 2 diabetes mellitus (T2DM), 3 enrolled exclusively patients with type 1 diabetes mellitus (T1DM [28, 40, 42]), 4 enrolled mixed T1DM/T2DM cohorts, and 2 did not specify diabetes type with sufficient clarity to classify. Age inclusion criteria varied across studies, ranging from ≥ 16 years [23] through 18–75 years [45] and 20–80 years [41, 48] to > 30 years [40]. Of the 26 studies, 22 framed OSA as the exposure and contributed to Arm 1 analyses, while 4 framed DR as the exposure [30, 32, 36, 41] and contributed to Arm 2 analyses.

In terms of confounder adjustment, two studies employed propensity-score matching (PSM), 21 employed multivariable regression, and three did not control for confounders. Eighteen studies adjusted for BMI as a covariate; 14 adjusted for diabetes duration. A complete list of adjustment variables for each study is provided in E-supplement 2. OSA was ascertained by polysomnography in the majority of studies; diagnostic criteria for both OSA and DR are provided in E-supplement 2.

### Methodological quality

Of the 26 included studies, 12 (46.2%) were rated good quality, 8 (30.8%) fair, and 6 (23.1%) poor on the Newcastle–Ottawa Scale (Table 4). Good-quality studies satisfied representativeness and non-exposed selection criteria, documented exposure ascertainment, and earned full comparability stars for confounder control. Recurrent limitations among studies rated as poor quality were absent or unclear reporting of DR diagnostic criteria, small sample size, and incomplete confounder adjustment.

**Table 4:** Methodological quality of the 26 included studies, assessed using the Newcastle–Ottawa Scale (NOS) with cohort or case-control versions applied as appropriate

Study	Selection			Comparability	Outcome			Rating	Reasons
	Representativeness of the exposed cohort	Selection of the non-exposed cohort	Ascertainment of exposure	Comparability of cohorts on the basis of the design or analysis controlled for confounders	Assessment of outcome	Was follow-up long enough for outcomes to occur	Adequacy of follow-up of cohorts		
Baba 2015	*	*	*	**	-	*	*	Fair	No mention of how DR was diagnosed
Ba-Ali 2023	*	*	*	**	*	*	*	Good	-
Chew 2019	*	*	*	**	*	*	*	Good	-
Du 2020	*	*	*	**	*	*	*	Good	-
El Ouardighi 2022	*	*	*	**	*	*	*	Good	-
Grauslund 2021	*	*	-	**	-	*	*	Poor	No mention of how DR and OSA were diagnosed.
Kaswan 2021	*	*	*	**	-	*	*	Fair	No mention of how DR was diagnosed
Nishimura 2015	*	*	*	**	*	*	*	Good	-
Zhang 2014	*	*	*	**	-	*	*	Fair	No mention of how DR was diagnosed
Yang 2022	-	*	*	-	-	*	*	Poor	No mention of how DR was diagnosed. Sample size was questionably small. No methods used for confounder adjustment.
Xue 2020	-	*	*	**	*	*	*	Fair	Sample size was questionably small
Pépin 2023	*	*	*	**	-	*	*	Fair	No mention of how DR was diagnosed
Rudrappa 2021	-	*	*	**	*	*	*	Fair	Sample size was questionably small
West 2010	*	*	*	**	*	*	*	Good	-
Manin 2014	*	*	*	**	*	*	*	Good	-
Chang 2018	-	*	*	**	-	*	*	Poor	No mention of how DR was diagnosed. Sample size was questionably small.
Banerjee 2013	*	*	*	**	-	*	*	Fair	No mention of how DR was diagnosed
Blegen 2021	*	*	*	**	*	*	*	Good	-
Chen 2020	-	*	*	*	-	*	*	Poor	No mention of how DR was diagnosed. Sample size was questionably small.
Adderley 2026	*	*	-	**	-	*	*	Poor	No mention of how DR and OSA were diagnosed.
Labban 2009	*	*	*	-	-	*	*	Poor	No methods used for confounder adjustment, no mention how DR were diagnosed
Storgaard 2014	*	*	*	**	*	*	*	Good	
Hermans 2013	*	*	*	-	*	*	*	Fair	No methods used for cofounder adjustment
Furukawa1 2013	*	*	*	**	*	*	*	Good	-
Altaf 2017	*	*	*	**	*	*	*	Good	-
Banghoej 2016	*	*	*	**	*	*	*	Good	-

Star ratings are presented across the selection, comparability, and outcome (or exposure) domains. Overall quality (good/fair/poor) was assigned using prespecified Agency for Healthcare Research and Quality (AHRQ) thresholds. Reasons for downgraded ratings are documented in the final column

*DR* diabetic retinopathy, *OSA* obstructive sleep apnea

### Arm 1: Studies framing OSA as the exposure for DR

#### Any DR, stratified by OSA severity

**Fig. 2:**
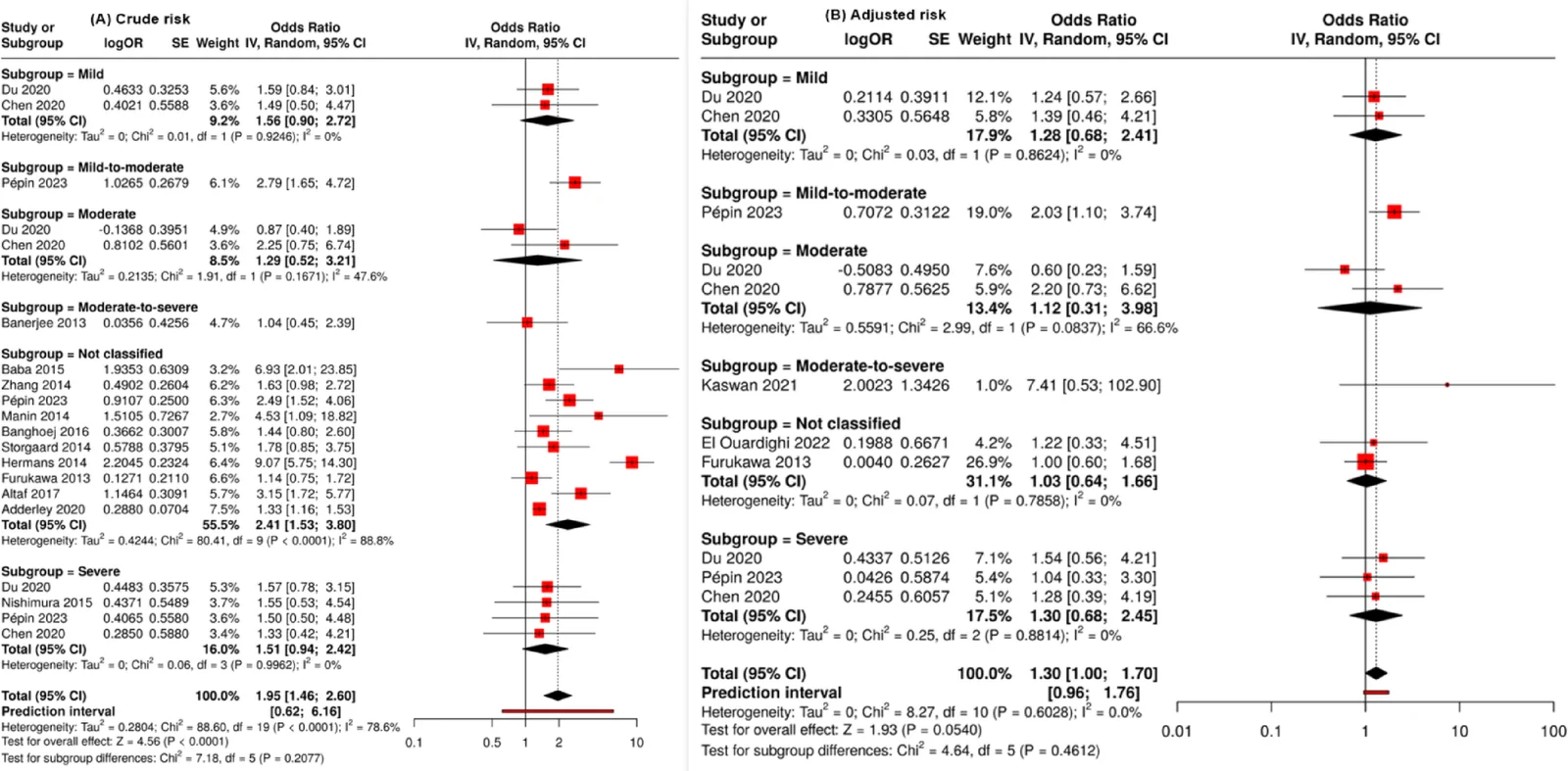
Pooled odds of any diabetic retinopathy (DR) in individuals with obstructive sleep apnea (OSA) versus those without OSA, stratified by OSA severity category (mild, moderate, severe, not classified). Panel **A** shows unadjusted estimates; Panel **B** shows confounder-adjusted estimates. The substantial attenuation from unadjusted (OR 2.41, 95% CI 1.53–3.80; Panel **A**) to adjusted (OR 1.03, 95% CI 0.64–1.66; Panel **B**) pooled estimates for OSA classified as a binary exposure is the principal Arm 1 finding. Random-effects meta-analysis using the DerSimonian–Laird estimator. Heterogeneity reported as I². GRADE certainty (very low/low/moderate/high) is indicated for each stratum. *OR* odds ratio, *CI* confidence interval, *GRADE* Grading of Recommendations Assessment, Development, and Evaluation

In unadjusted pooling (Fig. 2A), OSA classified as a binary exposure (severity not classified) was associated with higher odds of any DR (OR 2.41, 95% CI 1.53–3.80; 10 studies; I² = 88.8%; very low certainty). When stratified by AHI-based severity, unadjusted estimates were directionally consistent but did not reach statistical significance: mild OSA (OR 1.56, 95% CI 0.90–2.72; 2 studies; I² = 0%; low certainty), moderate OSA (OR 1.29, 95% CI 0.52–3.21; 2 studies; I² = 47.6%; very low certainty), and severe OSA (OR 1.51, 95% CI 0.94–2.42; 4 studies; I² = 0%; low certainty).

In confounder-adjusted pooling (Fig. 2B), the binary “OSA not classified” estimate attenuated substantially to OR 1.03 (95% CI 0.64–1.66; 2 studies; I² = 0%; low certainty). Adjusted estimates for mild, moderate, and severe OSA categories were similarly directionally consistent with the unadjusted pattern but did not reach statistical significance: mild OSA (OR 1.28, 95% CI 0.68–2.41; 2 studies; I² = 0%; low certainty), moderate OSA (OR 1.12, 95% CI 0.31–3.98; 2 studies; I² = 66.6%; very low certainty), and severe OSA (OR 1.30, 95% CI 0.68–2.45; 3 studies; I² = 0%; low certainty).

### DR stage-specific outcomes

**Fig. 3:**
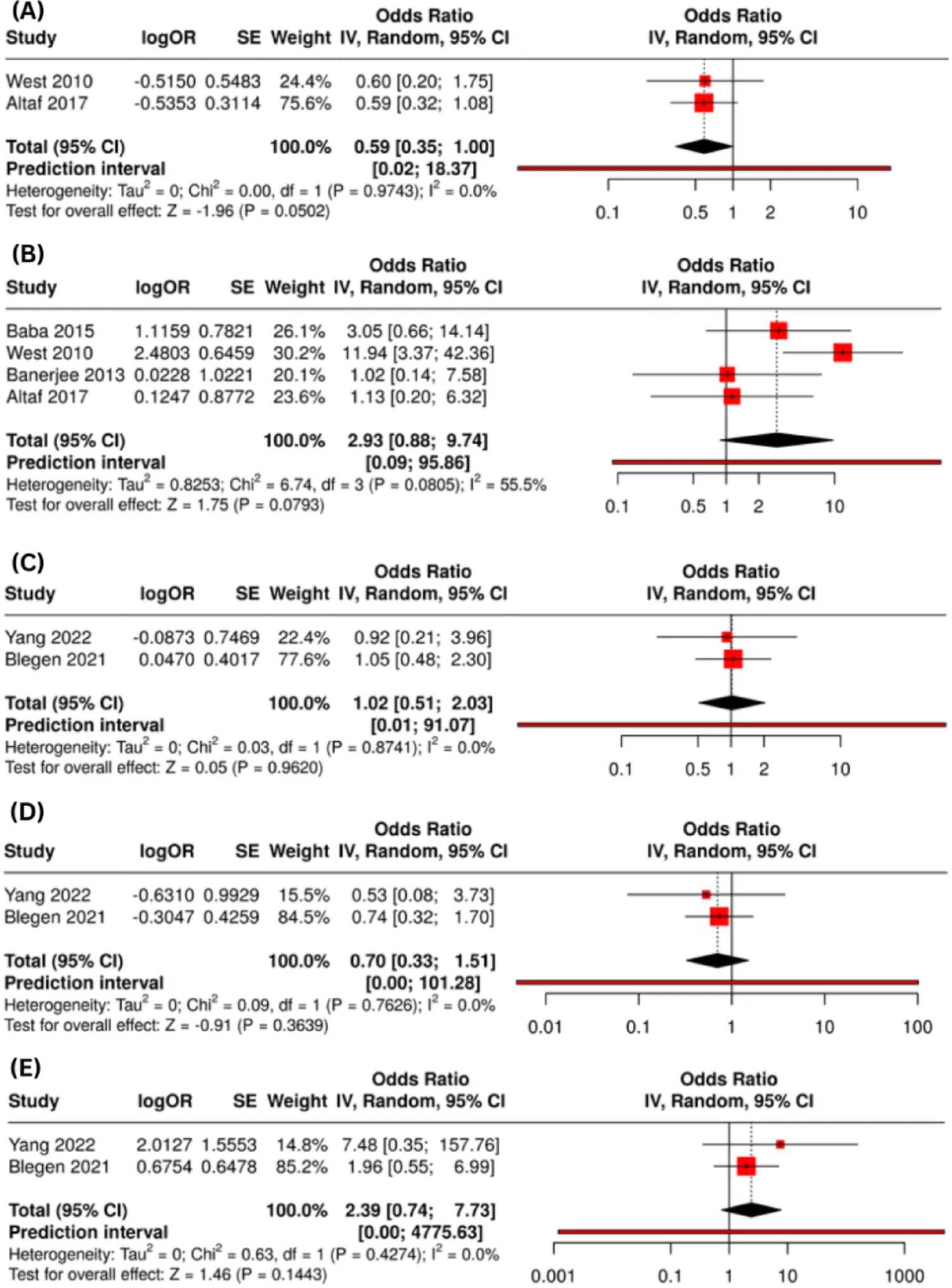
Pooled odds of stage-specific diabetic retinopathy (DR) outcomes in individuals with obstructive sleep apnea (OSA) versus those without OSA, with OSA classified as a binary exposure (severity not classified). Panel **A**, background DR; Panel **B**, mild non-proliferative DR (NPDR); Panel **C**, moderate NPDR; Panel D, severe NPDR; Panel E, overall NPDR. All estimates are unadjusted; severity-stratified data were not available at the stage level. Pooled estimates for proliferative DR (PDR), unadjusted and confounder-adjusted, are presented in E-supplements 5 and 6, respectively. Random-effects meta-analysis using the DerSimonian–Laird estimator. Heterogeneity reported as I². GRADE certainty indicated for each stratum. *OR* odds ratio, *CI* confidence interval

Stage-specific Arm 1 analyses used OSA classified as a binary exposure (severity not classified), as severity-stratified data were not available at the stage level (Fig. 3A–E). Unadjusted pooled estimates were: background DR (OR 0.59, 95% CI 0.35–1.00; 2 studies; I² = 0%; low certainty), mild NPDR (OR 1.02, 95% CI 0.51–2.03; 2 studies; I² = 0%; low certainty), moderate NPDR (OR 0.70, 95% CI 0.33–1.51; 2 studies; I² = 0%; low certainty), severe NPDR (OR 2.39, 95% CI 0.74–7.73; 2 studies; I² = 0%; low certainty), overall NPDR (OR 2.93, 95% CI 0.88–9.74; 4 studies; I² = 56%; very low certainty), and PDR (OR 1.83, 95% CI 0.79–4.26; 9 studies; I² = 88%; very low certainty, E-supplement 5). The PDR-adjusted pooled estimate, available from 2 studies, was OR 0.96 (95% CI 0.19–4.96; I² = 94.6%; very low certainty, E-supplement 6).

The “background DR” stratum was retained as the original authors reported it; we note that the two contributing studies labelled this category by their own conventions and it should not be interpreted as equivalent to mild NPDR (D1).

### Arm 2: Studies framing DR as the exposure for OSA

#### OSA severity as the outcome

When DR was treated as a binary exposure (severity not classified), pooled estimates for OSA severity outcomes were: mild OSA (OR 0.66, 95% CI 0.35–1.26; 2 studies; I² = 70.4%; very low certainty, E-supplement 8), moderate OSA (OR 0.99, 95% CI 0.49–2.00; 3 studies; I² = 70.2%; very low certainty, E-supplement 9), and severe OSA (OR 1.24, 95% CI 0.92–1.68; 4 studies; I² = 0%; low certainty, E-supplement 10). Estimates were directionally inverse for mild OSA, null for moderate OSA, and directionally positive but not statistically significant for severe OSA.

#### Overall OSA stratified by DR severity exposure

**Fig. 4:**
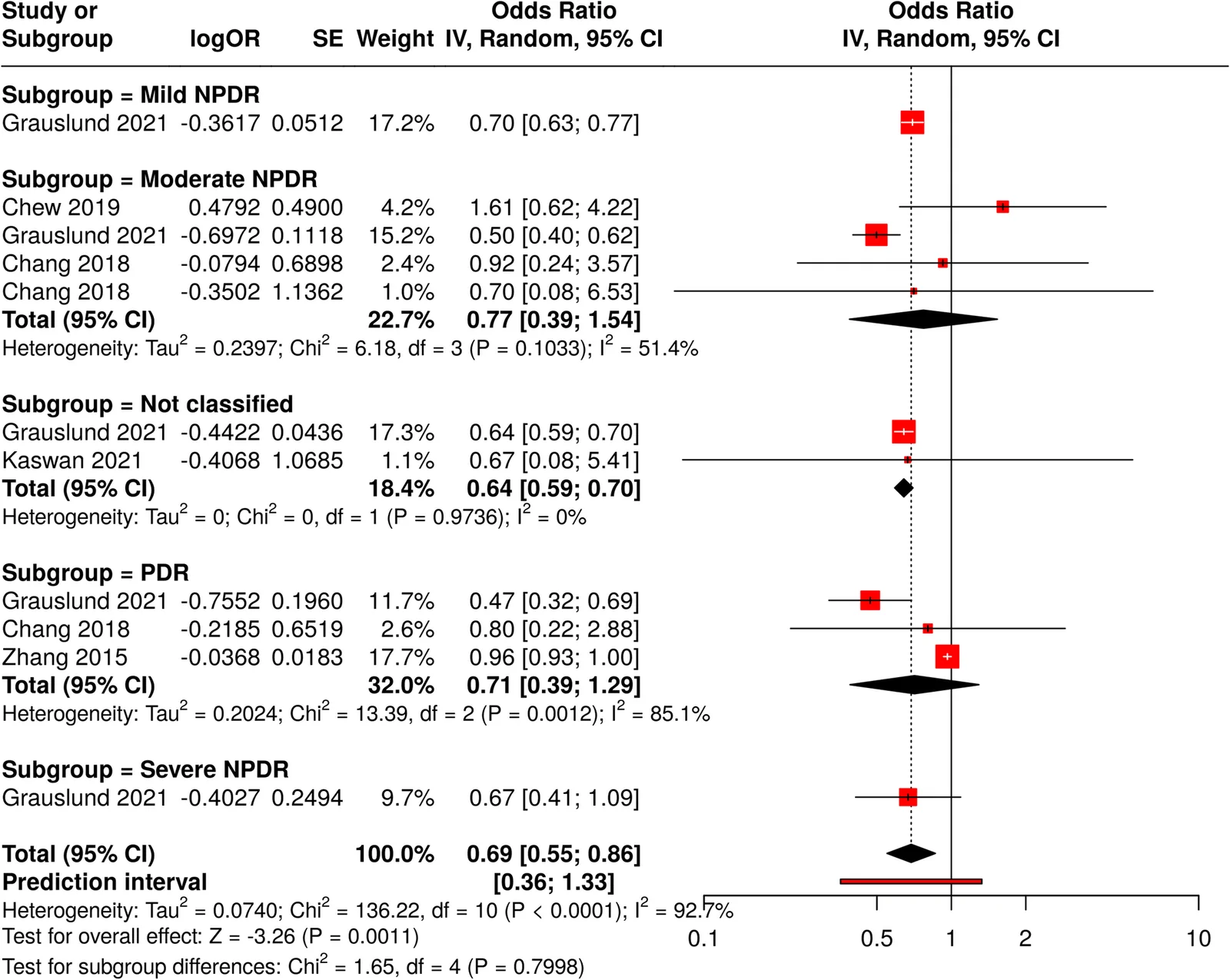
Pooled unadjusted odds of obstructive sleep apnea (OSA) in individuals with diabetic retinopathy (DR) versus those without DR, stratified by DR severity exposure: mild non-proliferative DR (NPDR), moderate NPDR, severe NPDR, proliferative DR (PDR), and DR not classified by severity. Strata in which only one study contributed an estimate (mild NPDR; severe NPDR) are indicated in the figure and reported as single-study estimates rather than pooled; the corresponding values are also reported narratively in the Results. Pooled estimates by OSA severity outcome (mild, moderate, severe), with DR classified as a binary exposure, are presented in E-supplements 7, 8, and 9, respectively. Random-effects meta-analysis using the DerSimonian–Laird estimator for strata with ≥ 2 contributing studies. Heterogeneity reported as I². GRADE certainty indicated for each stratum. *OR* odds ratio, *CI* confidence interval

In unadjusted pooling (Fig. 4), DR classified as a binary exposure (severity not classified) was associated with lower odds of OSA (OR 0.64, 95% CI 0.59–0.70; 2 studies; I² = 0%; low certainty). When DR was stratified by severity, unadjusted pooled estimates were available for moderate NPDR (OR 0.77, 95% CI 0.39–1.54; 4 studies; I² = 51.4%; very low certainty) and PDR (OR 0.71, 95% CI 0.39–1.29; 3 studies; I² = 85.1%; very low certainty). For mild NPDR (OR 0.70, 95% CI 0.63–0.77) and severe NPDR (OR 0.67, 95% CI 0.41–1.09), only one study contributed each, and these are reported as single-study estimates rather than pooled.

**Fig. 5:**
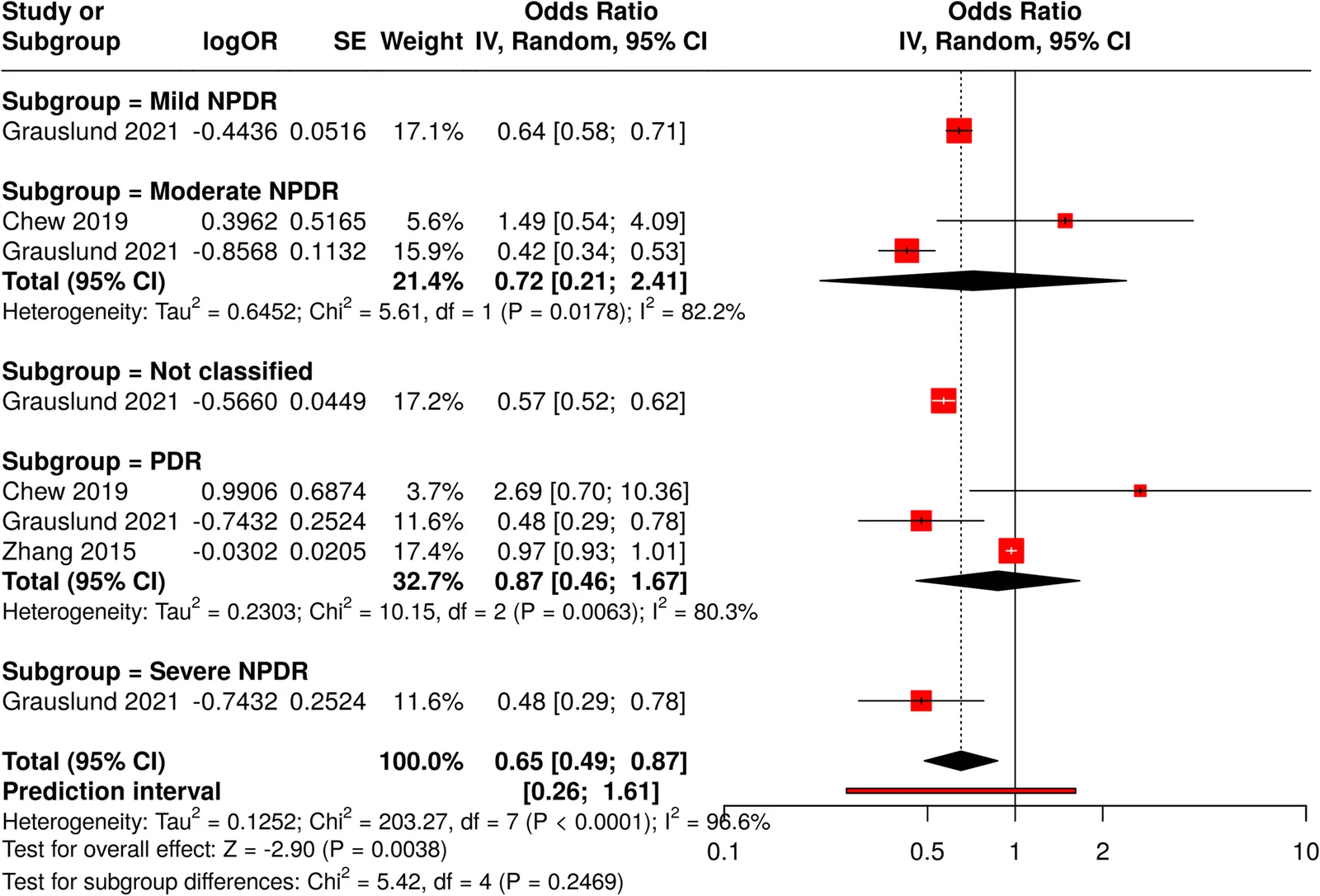
Pooled confounder-adjusted odds of obstructive sleep apnea (OSA) in individuals with diabetic retinopathy (DR) versus those without DR, stratified by DR severity exposure: mild non-proliferative DR (NPDR), moderate NPDR, severe NPDR, proliferative DR (PDR), and DR not classified by severity. Strata in which only one study contributed an estimate (mild NPDR; severe NPDR; DR not classified) are indicated in the figure and reported as single-study estimates rather than pooled; the corresponding values are also reported narratively in the Results. Random-effects meta-analysis using the DerSimonian–Laird estimator for strata with ≥ 2 contributing studies. Heterogeneity reported as I². GRADE certainty indicated for each stratum. *OR* odds ratio, *CI* confidence interval

In confounder-adjusted pooling (Fig. 5), estimates were available for moderate NPDR (OR 0.72, 95% CI 0.21–2.41; 2 studies; I² = 82.2%; very low certainty) and PDR (OR 0.87, 95% CI 0.46–1.67; 3 studies; I² = 80.3%; very low certainty). Single-study adjusted estimates were reported for mild NPDR (OR 0.64, 95% CI 0.58–0.71), severe NPDR (OR 0.48, 95% CI 0.29–0.78), and DR not classified (OR 0.57, 95% CI 0.52–0.62).

### Certainty of evidence

Certainty ratings for every pooled estimate are presented alongside the corresponding effect sizes in Tables 1 and 2. Across the 26 included studies, certainty ranged from low to very low; no stratum reached moderate certainty. The dominant grounds for downgrading were inconsistency (I² > 50% in several strata) and imprecision (wide confidence intervals from small numbers of contributing studies).

## Discussion

In this systematic review and meta-analysis of 26 observational studies including 706,105 individuals with diabetes, the association between OSA and DR proved sensitive to three structural features of the underlying evidence: the direction of the framed exposure–outcome relationship, the status of confounder adjustment, and the severity classification of both exposure and outcome. When studies were separated into those framing OSA as the exposure for DR (Arm 1) and those framing DR as the exposure for OSA (Arm 2), and when unadjusted and confounder-adjusted estimates were pooled separately within each arm, a coherent picture emerged that resolves several apparent contradictions in the prior literature. The unadjusted Arm 1 association between OSA and any DR (OR 2.41, 95% CI 1.53–3.80) attenuated substantially after confounder adjustment (OR 1.03, 95% CI 0.64–1.66). Inverse and null associations in Arm 2 reflect the different epidemiological question those studies were designed to answer, rather than a contradiction of the OSA-as-exposure literature. Severity-stratified Arm 1 estimates were directionally consistent with the overall finding but imprecise. Certainty of evidence was low to very low across most strata.

### Comparison with prior systematic reviews

Five prior systematic reviews and meta-analyses examined the OSA–DR relationship [1, 4, 9–11]. Each reported a positive overall pooled association, broadly consistent with the unadjusted Arm 1 finding in the present synthesis. However, prior reviews uniformly pooled studies framing OSA as the exposure together with studies framing DR as the exposure, and pooled crude with confounder-adjusted estimates within a single forest plot. The present synthesis advances on this work in five respects: (i) bidirectional separation of exposure direction; (ii) dual severity stratification of exposure and outcome within each arm; (iii) separate pooling of unadjusted and adjusted estimates; (iv) GRADE certainty rating at the per-stratum level; and (v) inclusion of seven studies not synthesised in earlier reviews. The substantive consequence of these refinements is that what previously appeared as a robust overall positive association is, on closer examination, a positive association in unadjusted Arm 1 estimates that attenuates to the null in confounder-adjusted analyses, accompanied by an inverse pattern in Arm 2 that should be interpreted as answering a different epidemiological question.

### Why do registry-based studies appear protective?

Two large registry-based studies [25, 36] reported inverse associations and are influential because of their precision. The bidirectional structure of the present analysis clarifies their contribution to the synthesised estimate. Grauslund 2021 [36] framed DR as the exposure and OSA as the outcome, and is therefore allocated to Arm 2, where its inverse direction is consistent with the wider Arm 2 pattern and answers a different epidemiological question from Arm 1. Ba-Ali 2023 [25] framed OSA as the exposure and contributes to Arm 1; its inverse direction within that arm warrants substantive interpretation.

Four non-mutually-exclusive structural mechanisms can account for inverse or attenuated registry-based estimates. First, surveillance and ascertainment bias: in national registries, individuals with a diagnosis of OSA receive systematic medical follow-up that typically includes glycaemic optimisation, lifestyle counselling, and structured ophthalmic screening, whereas the comparator group includes both true non-OSA participants and undiagnosed OSA, the latter often associated with lower-intensity diabetes care. Second, treatment effects: registry-based OSA cohorts contain a substantial fraction of individuals on continuous positive airway pressure (CPAP) therapy, which has been hypothesised to attenuate intermittent hypoxia and thereby reduce microvascular injury [9]. Third, healthy adherer effects: individuals who pursue and complete sleep evaluation in registry contexts tend to be more health-engaged, and the resulting selection is difficult to capture with available covariates. Fourth, differential outcome ascertainment: in screening registries, DR is identified by population-wide retinal photography applied uniformly, whereas in clinic-based studies DR ascertainment occurs in tertiary settings where the most severe phenotypes are over-represented; effect estimates in the latter are sensitive to referral patterns that have no analogue in registry data. The substantial attenuation observed when Arm 1 estimates are restricted to confounder-adjusted analyses (OR 2.41 → 1.03) suggests that confounding accounts for a substantial share of the previously reported positive association, with surveillance bias and healthy adherer effects providing plausible additional contributions in the registry-based subset.

### Biological plausibility

Despite the attenuation observed in confounder-adjusted analyses, the biological case for an OSA–DR relationship remains coherent. Intermittent hypoxia and re-oxygenation cycles drive oxidative stress, sympathetic activation, endothelial dysfunction, and inflammatory signalling that upregulate hypoxia-inducible factor–1α and vascular endothelial growth factor, promote leukostasis, and impair retinal microvascular autoregulation — mechanisms that could plausibly accelerate microangiopathy in diabetes [4]. These processes converge with diabetes-related pathways (advanced glycation, protein kinase C activation, nitrosative stress), providing shared molecular substrates for sleep-disordered breathing and microvascular complications [49]. Within diabetes cohorts, hypoxaemia metrics such as minimum nocturnal peripheral oxygen saturation (SpO₂) and the oxygen desaturation index correlate more closely with retinopathy than the apnea–hypopnea index (AHI) alone [9], suggesting that the dose, depth, and timing of hypoxia — rather than apnea frequency per se — may drive any genuine effect. Rapid eye movement (REM)-predominant OSA, characterised by longer apneas and deeper desaturations, has been particularly implicated [50]. The reconciliation of these biological considerations with the attenuated Arm 1 adjusted estimate is that mechanistic plausibility does not, on its own, establish a clinically detectable population-level association after confounder adjustment; the present results indicate that any true effect, if present, is smaller than unadjusted estimates suggest.

### Stage-specific findings within Arm 1

Stage-specific Arm 1 estimates were directionally aligned with the overall Arm 1 finding (severe NPDR OR 2.39, 95% CI 0.74–7.73; overall NPDR OR 2.93, 95% CI 0.88–9.74; PDR-unadjusted OR 1.83, 95% CI 0.79–4.26) but imprecise. Three structural factors limit the strength of inference at the stage level. First, sparse strata: most contributing studies reported severity-stratified outcomes only for the binary “any DR” endpoint and reported stage-specific outcomes only with OSA as a binary exposure, leaving 2–9 studies per stage-specific stratum. Second, differential adjustment by stage: studies contributing to mild and moderate NPDR strata are predominantly clinic-based cross-sectional reports in which the comparator group includes participants with high background metabolic risk; when these covariates are not fully adjusted at the stage level, estimates are biased toward the null. Third, the “background DR” stratum was retained as the original authors reported it; the two contributing studies labelled this category by their own conventions, and the inverse direction in this small stratum should be regarded as hypothesis-generating only.

## Clinical implications

For practising ophthalmologists, three clinical messages follow from the present synthesis. First, OSA evaluation in patients with diabetes should be considered on the basis of clinical suspicion and in the context of advanced or rapidly progressing DR, but the evidence base does not currently support routine OSA screening as a strategy to prevent or modify DR. The substantial attenuation of the OSA–DR association after confounder adjustment indicates that BMI, age, diabetes duration, and metabolic comorbidities account for a substantial share of the unadjusted signal. Second, polysomnography-confirmed OSA, rather than questionnaire-based or administrative diagnosis, is the preferred ascertainment standard; observational data suggest that nocturnal hypoxaemia metrics (minimum SpO₂, oxygen desaturation index, REM-AHI) may be more strongly associated with retinopathy than AHI alone [9, 50], and where available these metrics warrant inclusion in clinical decision-making. Third, causal inferences should not be drawn from the present synthesis. The certainty of evidence is low to very low across all strata, and definitive evidence that treating OSA prevents or slows DR awaits prospective trials.

### Strengths and limitations

Strengths of the present work include the bidirectional analytical structure, dual severity stratification, separate pooling of unadjusted and adjusted estimates, per-stratum GRADE assessment, comprehensive search supplemented by re-screening of five prior systematic reviews, and an expanded evidence base of 26 studies. Limitations are substantial and warrant explicit discussion.

First, temporality was clearly ascertained in only 11 of 26 studies; the remainder were cross-sectional or retrospective designs in which temporal precedence of OSA over DR (or vice versa) was either vague or not documented. Allocation to Arm 1 versus Arm 2 was therefore based on the original authors’ analytical framing rather than on a definitive temporal sequence, and we cannot exclude that some studies allocated to Arm 1 included prevalent DR cases. Second, diabetes type was heterogeneous across studies: 17 studies enrolled exclusively T2DM populations, 3 enrolled exclusively T1DM, 4 enrolled mixed cohorts, and 2 did not specify; the bulk of the synthesised evidence therefore reflects T2DM, and conclusions for T1DM rest on a small subset. Third, age inclusion criteria varied substantially across studies (≥ 16, 18–75, 20–80, ≥ 21, >30 years), introducing population heterogeneity that is not fully addressed by adjustment for mean age. Fourth, diagnostic criteria for both OSA and DR were variable: while most studies used polysomnography for OSA, AHI thresholds and DR grading conventions (ICDR, ETDRS, author-defined) were not uniform, and several studies did not document DR diagnostic criteria at all. Fifth, residual confounding cannot be excluded. Although most included studies adjusted for major covariates including age, BMI, and diabetes duration, fewer adjusted for HbA1c, hypertension, dyslipidaemia, smoking, or socioeconomic status; surveillance bias and healthy adherer effects in registry-based studies are particularly difficult to capture with available covariates. Sixth, multivariable meta-regression on diabetes type, BMI, age, and diabetes duration was not feasible because most stratified analyses contained 2–9 studies (below the ≥ 10 studies-per-covariate threshold for reliable meta-regression) and because aggregate-level meta-regression on individual-level confounders is subject to ecological fallacy. Seventh, publication bias could not be formally evaluated in most strata because of low study counts, and funnel-plot tests in the strata with ≥ 10 studies would have violated the independence assumption because individual studies contributed multiple effect estimates. Eighth, only aggregate study-level data were synthesised; individual-participant data were not requested.

### Future research

Three priorities follow. First, prospective cohorts with ascertained temporality are needed in which incident DR is the outcome of interest in OSA-positive and OSA-negative cohorts at baseline, ideally with polysomnographic OSA confirmation and standardised DR grading. Second, individual-participant data meta-analyses incorporating diabetes type, BMI, age, diabetes duration, HbA1c, and hypoxaemia metrics as continuous covariates would address both the multivariable risk-factor question raised by reviewers and the limitations of aggregate-level meta-regression. Third, randomised trials evaluating whether CPAP therapy modifies the trajectory of incident or progressive DR would provide the strongest evidence for or against a causal OSA–DR relationship, and would clarify whether OSA evaluation has a place in the prevention of microvascular complications in diabetes.

## Conclusions

This bidirectional meta-analysis indicates that the association between OSA and DR is sensitive to the direction of the framed exposure–outcome relationship and to confounder adjustment. The unadjusted Arm 1 association attenuates substantially after adjustment, and Arm 2 estimates are inverse or null and reflect a different epidemiological question. Mechanistic plausibility for an OSA–DR effect remains coherent, but certainty of evidence is low to very low across all strata. Polysomnography-confirmed prospective cohorts with uniform DR grading, individual-participant-level adjustment for metabolic confounders, and randomised evaluation of CPAP therapy are needed to clarify the direction and magnitude of the relationship and to establish whether OSA evaluation should have a defined role in the prevention of diabetic retinopathy.

## Supplementary Information

Below is the link to the electronic supplementary material.

Supplementary Material 1. E-supplement 1: Complete database search strategy for PubMed, Scopus, Web of Science, and Google Scholar, including controlled vocabulary terms, text words, Boolean operators, and the broadened term set used to capture diabetic retinopathy data reported only within full texts or supplementary materials. Search dates: database inception to July 13, 2025.

Supplementary Material 2. E-supplement 2: Diagnostic criteria and confounder-adjustment details for the 26 included studies. Reported by study, the supplement provides: (a) the diagnostic criterion used to ascertain obstructive sleep apnea (OSA), including polysomnography parameters and apnea–hypopnea index (AHI) thresholds where reported; (b) the diagnostic criterion used to ascertain diabetic retinopathy (DR), including grading conventions (International Clinical Diabetic Retinopathy [ICDR], Early Treatment Diabetic Retinopathy Study [ETDRS], or author-defined); (c) the confounder-adjustment approach (multivariable regression, propensity-score matching, or unadjusted); and (d) the complete list of covariates entered into the adjusted analyses where reported.

Supplementary Material 3. E-supplement 3: Calculated odds ratios (ORs) and 95% confidence intervals derived from 2×2 contingency tables for studies in Arm 1 (obstructive sleep apnea [OSA] as the exposure, diabetic retinopathy [DR] as the outcome). For studies that reported crude associations as 2×2 cell counts rather than as a precomputed OR, event and non-event counts in OSA-exposed and unexposed groups are presented alongside the corresponding calculated OR with 95% confidence interval. Calculations were performed in R. The values reported in this supplement are those entered into the Arm 1 unadjusted pooled analyses presented in Table 1 and Figures 2A and 3A–E.

Supplementary Material 4. E-supplement 4: Calculated odds ratios (ORs) and 95% confidence intervals derived from 2×2 contingency tables for studies in Arm 2 (diabetic retinopathy [DR] as the exposure, obstructive sleep apnea [OSA] as the outcome). For studies that reported crude associations as 2×2 cell counts rather than as a precomputed OR, event and non-event counts in DR-exposed and unexposed groups are presented alongside the corresponding calculated OR with 95% confidence interval. Calculations were performed in R. The values reported in this supplement are those entered into the Arm 2 unadjusted pooled analyses presented in Table 2, Figure 4, and E-supplements 7–9.

E-supplement 5: Pooled unadjusted odds of proliferative diabetic retinopathy (PDR) in individuals with obstructive sleep apnea (OSA) versus those without OSA, with OSA classified as a binary exposure (severity not classified). Forest plot with study-level effect estimates, weights, pooled effect, and heterogeneity statistic. This figure presents Arm 1 PDR data referenced in Table 1 and the Results. Random-effects meta-analysis using the DerSimonian–Laird estimator. OR, odds ratio; CI, confidence interval.

Supplementary Material 5

E-supplement 6: Pooled confounder-adjusted odds of proliferative diabetic retinopathy (PDR) in individuals with obstructive sleep apnea (OSA) versus those without OSA, with OSA classified as a binary exposure (severity not classified). Forest plot with study-level effect estimates, weights, pooled effect, and heterogeneity statistic. This figure presents Arm 1 PDR data referenced in Table 1 and the Results. Random-effects meta-analysis using the DerSimonian–Laird estimator. OR, odds ratio; CI, confidence interval.

Supplementary Material 6

E-supplement 7: Pooled odds of mild obstructive sleep apnea (OSA) (apnea–hypopnea index 5 to <15 events/h) in individuals with diabetic retinopathy (DR) versus those without DR, with DR classified as a binary exposure (severity not classified). Forest plot with study-level effect estimates, weights, pooled effect, and heterogeneity statistic. This figure presents Arm 2 mild-OSA-outcome data referenced in Table 2 and the Results. Random-effects meta-analysis using the DerSimonian–Laird estimator. OR, odds ratio; CI, confidence interval.

Supplementary Material 7

E-supplement 8: Pooled odds of moderate obstructive sleep apnea (OSA) (apnea–hypopnea index 15 to <30 events/h) in individuals with diabetic retinopathy (DR) versus those without DR, with DR classified as a binary exposure (severity not classified). Forest plot with study-level effect estimates, weights, pooled effect, and heterogeneity statistic. This figure presents Arm 2 moderate-OSA-outcome data referenced in Table 2 and the Results. Random-effects meta-analysis using the DerSimonian–Laird estimator. OR, odds ratio; CI, confidence interval.

Supplementary Material 8

E-supplement 9: Pooled odds of severe obstructive sleep apnea (OSA) (apnea–hypopnea index ≥30 events/h) in individuals with diabetic retinopathy (DR) versus those without DR, with DR classified as a binary exposure (severity not classified). Forest plot with study-level effect estimates, weights, pooled effect, and heterogeneity statistic. This figure presents Arm 2 severe-OSA-outcome data referenced in Table 2 and the Results. Random-effects meta-analysis using the DerSimonian–Laird estimator. OR, odds ratio; CI, confidence interval.

Supplementary Material 9
