# Supplementary material for: Bidirectional association between obstructive sleep apnea and diabetic retinopathy: a severity-stratified systematic review and meta-analysis with GRADE assessment
Source: Acta Diabetol. 2026 Jun 23;63(12):1997–2013. doi: 10.1007/s00592-026-02732-2 (PMC13769771; doi:10.1007/s00592-026-02732-2)
Supplement: Supplementary file 1 [file 592_2026_2732_MOESM1_ESM.docx]

**E-supplement 3.** Calculated odds ratios from 2×2 contingency tables for studies in Arm 1 (obstructive sleep apnea as the exposure, diabetic retinopathy as the outcome).

| **#** | **Study** | **Outcome (DR)** | **Exposure (OSA)** | **Experimental** | **Control** | **OR** | **95% CI** |
| --- | --- | --- | --- | --- | --- | --- | --- |
| 1 | Baba 2015 | NC | NC | 11 / 20 | 6 / 40 | 6.93 | 2.011 – 23.853 |
| 2 | Baba 2015 | PDR | NC | 4 / 20 | 3 / 40 | 3.08 | 0.618 – 15.390 |
| 3 | Baba 2015 | NPDR | NC | 7 / 20 | 3 / 20 | 3.05 | 0.659 – 14.137 |
| 4 | Ba-Ali 2023 | PDR | NC | 231 / 13279 | 17210 / 519549 | 0.52 | 0.453 – 0.589 |
| 5 | Nishimura 2015 | NC | Severe | 6 / 17 | 31 / 119 | 1.55 | 0.528 – 4.540 |
| 6 | Zhang 2014 | NC | NC | 68 / 310 | 24 / 162 | 1.62 | 0.970 – 2.691 |
| 7 | Zhang 2014 | PDR | NC | 13 / 310 | 4 / 162 | 1.73 | 0.555 – 5.391 |
| 8 | Yang 2022 | Mild NPDR | NC | 11 / 17 | 10 / 15 | 0.92 | 0.212 – 3.961 |
| 9 | Yang 2022 | Moderate NPDR | NC | 2 / 17 | 0 / 15 | 0.53 | 0.076 – 3.725 |
| 10 | Yang 2022 | Severe NPDR | NC | 3 / 17 | 0 / 15 | 7.48 | 0.355 – 157.761 |
| 11 | Yang 2022 | PDR | NC | 0 / 17 | 0 / 15 | — | NA – NA |
| 12 | Pépin 2023 | NC | Mild-to-moderate | 24 / 95 | 70 / 648 | 2.79 | 1.651 – 4.719 |
| 13 | Pépin 2023 | NC | Severe | 4 / 26 | 70 / 648 | 1.50 | 0.503 – 4.482 |
| 14 | Pépin 2023 | NC | NC | 28 / 121 | 70 / 648 | 2.49 | 1.523 – 4.058 |
| 15 | Rudrappa 2021 | PDR | NC | 4 / 16 | 0 / 17 | 12.60 | 0.621 – 255.762 |
| 16 | West 2010 | Background | NC | 5 / 28 | 24 / 90 | 0.60 | 0.204 – 1.750 |
| 17 | West 2010 | NPDR | NC | 10 / 28 | 4 / 90 | 11.94 | 3.368 – 42.360 |
| 18 | West 2010 | PDR | NC | 0 / 28 | 0 / 90 | — | NA – NA |
| 20 | Manin 2014 | PDR | NC | 23 / 31 | 11 / 36 | 6.53 | 2.236 – 19.096 |
| 21 | Banerjee 2013 | NC | Moderate-to-severe | 18 / 46 | 18 / 46 | 1.04 | 0.450 – 2.386 |
| 22 | Banerjee 2013 | NPDR | NC | 2 / 46 | 2 / 47 | 1.02 | 0.138 – 7.584 |
| 23 | Banerjee 2013 | PDR | NC | 1 / 46 | 3 / 47 | 0.33 | 0.033 – 3.254 |
| 24 | Blegen 2021 | Mild NPDR | NC | 18 / 32 | 65 / 118 | 1.05 | 0.477 – 2.303 |
| 25 | Blegen 2021 | Moderate NPDR | NC | 10 / 32 | 45 / 118 | 0.74 | 0.320 – 1.699 |
| 26 | Blegen 2021 | Severe NPDR | NC | 4 / 32 | 8 / 118 | 1.96 | 0.552 – 6.994 |
| 27 | Banghoej 2016 | PDR | NC | 18 / 92 | 10 / 107 | 2.36 | 1.029 – 5.412 |
| 28 | Banghoej 2016 | NC | NC | 29 / 92 | 35 / 107 | 0.95 | 0.521 – 1.732 |
| 29 | Storgaard 2014 | PDR | NC | 2 / 72 | 3 / 108 | 1.00 | 0.163 – 6.138 |
| 30 | Storgaard 2014 | NC | NC | 18 / 72 | 17 / 108 | 1.78 | 0.848 – 3.753 |
| 31 | Hermans 2014 | NC | NC | 53 / 129 | 49 / 686 | 9.07 | 5.749 – 14.296 |
| 32 | Furukawa 2013 | NC | NC | 56 / 233 | 61 / 280 | 1.14 | 0.751 – 1.717 |
| 33 | Altaf 2017 | Background | NC | 31 / 147 | 26 / 83 | 0.59 | 0.318 – 1.078 |
| 34 | Altaf 2017 | NPDR | NC | 4 / 147 | 2 / 83 | 1.13 | 0.203 – 6.321 |
| 35 | Altaf 2017 | PDR | NC | 4 / 147 | 3 / 83 | 0.75 | 0.163 – 3.417 |
| 36 | Altaf 2017 | NC | NC | 71 / 147 | 19 / 83 | 3.15 | 1.717 – 5.767 |
| 37 | Adderley 2020 | NC | NC | 320 / 3667 | 699 / 10450 | 1.33 | 1.162 – 1.531 |

*OR: odds ratio; CI: confidence interval; NA: not available (zero cells in both arms; contributes no information to the pooled estimate).*
