# Supplementary material for: Bidirectional association between obstructive sleep apnea and diabetic retinopathy: a severity-stratified systematic review and meta-analysis with GRADE assessment
Source: Acta Diabetol. 2026 Jun 23;63(12):1997–2013. doi: 10.1007/s00592-026-02732-2 (PMC13769771; doi:10.1007/s00592-026-02732-2)
Supplement: Supplementary file 2 [file 592_2026_2732_MOESM2_ESM.docx]

**E-supplement 4.** Calculated odds ratios from 2×2 contingency tables for studies in Arm 2 (diabetic retinopathy as the exposure, obstructive sleep apnea as the outcome).

| **#** | **Study** | **Exposure (DR)** | **Outcome (OSA)** | **Experimental** | **Control** | **OR** | **95% CI** |
| --- | --- | --- | --- | --- | --- | --- | --- |
| 1 | Xue 2020 | NC | Mild | 50 / 110 | 170 / 353 | 0.90 | 0.584 – 1.378 |
| 2 | Laaban 2009 | NC | Mild | 49 / 103 | 74 / 112 | 0.47 | 0.269 – 0.808 |
| 3 | Chang 2018 | Mild NPDR | Mild-to-moderate | 34 / 72 | 45 / 97 | 1.03 | 0.561 – 1.905 |
| 4 | Chang 2018 | PDR | Mild-to-moderate | 20 / 79 | 45 / 97 | 0.39 | 0.205 – 0.747 |
| 5 | Chang 2018 | NC | Mild-to-moderate | 58 / 220 | 45 / 97 | 0.41 | 0.251 – 0.682 |
| 6 | Chew 2019 | Moderate NPDR | Moderate | 16 / 29 | 21 / 63 | 2.46 | 1.001 – 6.054 |
| 7 | Xue 2020 | NC | Moderate | 28 / 110 | 100 / 353 | 0.86 | 0.531 – 1.407 |
| 8 | Laaban 2009 | NC | Moderate | 30 / 57 | 74 / 112 | 0.57 | 0.298 – 1.094 |
| 9 | Kaswan 2021 | NC | Moderate-to-severe | 6 / 348 | 1 / 14 | 0.23 | 0.026 – 2.034 |
| 10 | Kaswan 2021 | Severe NPDR | Moderate-to-severe | 6 / 348 | 1 / 14 | 0.23 | 0.026 – 2.034 |
| 11 | Chew 2019 | Moderate NPRD | NC | 21 / 29 | 39 / 63 | 1.62 | 0.618 – 4.219 |
| 12 | Kaswan 2021 | NC | NC | 17 / 348 | 1 / 14 | 0.67 | 0.082 – 5.406 |
| 13 | Chang 2018 | Moderate NPDR | NC | 68 / 72 | 92 / 97 | 0.92 | 0.239 – 3.570 |
| 14 | Chang 2018 | Moderate NPDR | NC | 13 / 14 | 92 / 97 | 0.71 | 0.076 – 6.532 |
| 15 | Chang 2018 | PDR | NC | 74 / 79 | 92 / 97 | 0.80 | 0.224 – 2.884 |
| 16 | Nishimura 2015 | NC | Severe | 6 / 37 | 11 / 99 | 1.55 | 0.528 – 4.540 |
| 17 | Xue 2020 | NC | Severe | 31 / 110 | 76 / 353 | 1.43 | 0.879 – 2.327 |
| 18 | Chang 2018 | NC | Severe | 111 / 220 | 47 / 97 | 1.08 | 0.672 – 1.747 |
| 20 | Laaban 2009 | NC | Severe | 21 / 31 | 74 / 112 | 1.08 | 0.462 – 2.519 |

*OR: odds ratio; CI: confidence interval. Identical rows for a single study reflect that study's contribution to multiple severity-stratified subgroup analyses with the same underlying 2×2 data.*
