# Supplementary material for: Bidirectional association between obstructive sleep apnea and diabetic retinopathy: a severity-stratified systematic review and meta-analysis with GRADE assessment
Source: Acta Diabetol. 2026 Jun 23;63(12):1997–2013. doi: 10.1007/s00592-026-02732-2 (PMC13769771; doi:10.1007/s00592-026-02732-2)
Supplement: Supplementary file 3 [file 592_2026_2732_MOESM3_ESM.docx]

**E-supplement 1.** The search query employed in the database search

| No. | Search query | Results |
| --- | --- | --- |
| PubMed | | |
| #1 | “sleep apnea” OR “sleep apnoea” OR “obstructive sleep” OR OSAHS OR “sleep hypopnea” OR “sleep hypopnoea” OR “sleep-disordered breathing” OR “sleep disordered breathing” OR "Sleep Apnea, Obstructive"[Mesh] OR "Sleep Apnea Syndromes"[Mesh] | 65939 |
| #2 | “diabetic retinopathy” OR "Diabetic Retinopathy"[Mesh] | 45643 |
| #4 | #1 AND #2 | 96 |
| Scopus | | |
| #1 | ALL (“sleep apnea”) OR ALL (“sleep apnoea”) OR ALL (“obstructive sleep”) OR ALL (OSAHS) OR ALL (“sleep hypopnea”) OR ALL (“sleep hypopnoea”) OR ALL (“sleep-disordered breathing”) OR ALL (“sleep disordered breathing”) | 207618 |
| #2 | ALL (“diabetic retinopathy”) | 185609 |
| #3 | #1 AND #2 | 1154 |
| Web of Science | | |
| #1 | ALL=“sleep apnea” OR ALL=“sleep apnoea” OR ALL=“obstructive sleep” OR ALL=OSAHS OR ALL=“sleep hypopnea” OR ALL=“sleep hypopnoea” OR ALL=“sleep-disordered breathing” OR ALL=“sleep disordered breathing” | 82903 |
| #2 | ALL=“diabetic retinopathy” | 46639 |
| #3 | #1 AND #2 | 116 |
| Google Scholar | | |
| With all of the words | diabetic retinopathy | - |
| With the exact phrase | sleep apnea | - |
| With at least one of the words | - | - |
| Total | - | 200 |
