# Supplementary material for: Bidirectional association between obstructive sleep apnea and diabetic retinopathy: a severity-stratified systematic review and meta-analysis with GRADE assessment
Source: Acta Diabetol. 2026 Jun 23;63(12):1997–2013. doi: 10.1007/s00592-026-02732-2 (PMC13769771; doi:10.1007/s00592-026-02732-2)
Supplement: Supplementary file 4 [file 592_2026_2732_MOESM4_ESM.docx]

**E-supplement 2.** A descriptive summary of confounding adjustment method and variable and the diagnostic criteria for diabetic retinopathy and obstructive sleep apnea

| Study | Exposure | Temporality ascertainment | OSA diagnosis | DR diagnosis | Confounding adjustment | Adjusted variables |
| --- | --- | --- | --- | --- | --- | --- |
| Chew 2019 | DR | Not ascertained | Diagnosed through polysomnography (Any: AHI >= 5; moderate: AHI >= 15) | Retinal images were analyzed to identify any characteristic lesions as defined by the Early Treatment for DR Study (ETDRS) severity scale: microaneurysms, hemorrhages, cotton wool spots, intraretinal microvascular abnormalities, hard exudates, venous bleeding, and new vessels. Scoring was based on the modified Airlie House Classification System and the eye with worse retinopathy score was used for analysis. “Moderate DR” was defined as level 43 and above as this level was thought to be more diabetes-specific; while VTDR was defined as the presence of severe non-proliferative DR, proliferative DR or clinically significant macular edema. | Regression | Age and gender. |
| Grauslund 2021 | DR | Ascertained (index date) | NR | NR | Regression | Age and gender. |
| Nishimura 2015 | DR | Ascertained (prior exposure of OSA before DR) | Polysomnography (AHI) score of ≥ 30/h | Optic fundi were examined using indirect ophthalmoscope or retinal photographs. | Regression | age, sex, body mass index, eGFR, HbA1c, triglyceride, total cholesterol, high density lipoprotein (HDL), cholesterol, LDL cholesterol, non-HDL cholesterol, urine protein, creatinine ratio, duration of diabetes, history of hypertension, history of CVD, smoking, percentage of time spent with SO2<90, apnea hypopnea index, 4% oxygen desaturation index, 3% oxygen desaturation index, minimum SO2, and medications. |
| Chang 2018 | DR | Not ascertained | Polysomnography: (AHI) >5 events/h (mild), 15–30 events/h (moderate) and AHI ≥30 (severe). | NR | Regression | Age, sex, ethnicity, BMI, DM duration, HbA1c, smoking history, presence of hypertension, dyslipidemia, and carotid stenosis. |
| Baba 2015 | OSA | Not ascertained | Diagnosed through polysomnography (AHI >= 5) | NR | Matching | Age and duration of diabetes |
| Ba-Ali 2023 | OSA | Ascertained | Diagnosed through polysomnography (AHI >= 5) | Using the Health Care Classification System (SKS) for retinal photocoagulation laser including pan-retinal photocoagulation and local retinal photocoagulation (KCKC10 and KCKC15) and anti-VEGF (BOHJ19B) in DNPR. | Regression | Age, sex and marital status. |
| Du 2020 | OSA | Not ascertained | Polysomnography: (AHI) >5 events/h (mild), 15–30 events/h (moderate) and AHI ≥30 (severe). | The presence of DR that encompassed simple, non-proliferative, or proliferative retinopathy was diagnosed by an experienced ophthalmologist who evaluated the optical fundi by using ophthalmoscopy or retinal photography. | Regression | Age, sex, current smoking, regular drinking, BMI, systolic and diastolic BP, diabetes duration, OGLA use, Angiotensin-converting enzyme inhibitor/Angiotension receptor antagonist (ACEI/ARB) use, statin use, triglyceride, total cholesterol, HDL-cholesterol, and sleep time. |
| El Ouardighi 2022 | OSA | Ascertained (excluded prior OSA) | Polysomnography: (AHI) >5 events/h (mild), 15–30 events/h (moderate) and AHI ≥30 (severe). | “Mild non-proliferative retinopathy” (NPDR), characterized by the presence of a few microaneurysms. “Moderate NPDR” is characterized by the presence of microaneurysms, intraretinal hemorrhages, or venous beading. The diagnosis of severe NPDR is based on the 4:2:1 rule of the ETDRS, meaning that it is present if hemorrhages are present in all 4 quadrants, if 2 quadrants or more have venous beading (VB), if one or more quadrant has intra retinal microvascular abnormalities (IRMA). The “proliferative diabetic retinopathy” (PDR) is characterized by neovascularization of the disc, neovascularization of the retina, neovascularization of the iris, neovascularization of the angle, vitreous hemorrhage, or tractional retinal detachment. | Regression | age, BMI, T2D duration, HbA1c, systolic BP, and the presence of OSA (OAHI≥5/ hour of sleep on PSG), ODI, OAHI, OAI, time spent<90% oxy gen saturation, lowest oxygen saturation, and mean apnea duration. |
| Kaswan 2021 | OSA | Not ascertained | Polysomnography: (AHI) >5 events/h (mild), 15–30 events/h (moderate) and AHI ≥30 (severe). Any OSA diagnosed as AHI >5 events/h | NR | Regression | Age, gender, duration of diabetes, glycosylated hemoglobin (HBA1c), anemia, CHD, hypertension, dyslipidemia (LDL/HDL ratio), history of smoking/alcoholism. |
| Zhang et 2014 | OSA | Not ascertained | Polysomnography (AHI) score of ≥5/h | NR | Regression | age, sex, BMI, ESS, habitual snoring, snoring with apnea, duration of diabetes, and HbA1c. |
| Yang 2022 | OSA | Not ascertained | Polysomnography (AHI) score of ≥ 15/h | NR | NA | NA |
| Xue 2020 | OSA | Not ascertained | Polysomnography | E11. 319 | Regression | age, sex, race, body mass index (BMI), smoking status, alcohol consumption, family history of diabetes, diabetes duration, antidiabetic treatment, antihypertensive agents, lipid-lowering therapy, HbA1c, (MAP), triglycerides, (LDL-C), (HDL-C), eGFR. |
| Pépin 2023 | OSA | Not ascertained | No sleep apnoea was defined as an ODI of <15 events/h, mild-to-moderate sleep apnoea as an ODI of 15 to <30 events/h, and severe sleep apnoea as an ODI of ≥30 events/h. | NR | Regression | Age, sex and body mass index (BMI). |
| Rudrappa 2015 | OSA | Not ascertained | Polysomnography: (AHI) >5 events/h (mild), 15–30 events/h (moderate) and AHI ≥30 (severe). | Digital photography of the retina and systematic image grading by optometrists and/or ophthalmologist. | Matching | All clinical and biochemical parameters were equally matched between OSA and non-OSA patients except for Epworth and the AHI scores. |
| West 2010 | OSA | Not ascertained | 1. more than 10, > 4% oxygen saturation (SaO2) dips⁄ h on oximetry. 2. Fewer than 10 > 4% SaO2 dips⁄ h, but had an oxygen saturation tracing consistent with a diagnosis of OSA when viewed by an expert. | The grading structure involves using the overall grades recommended by the English National Screening Programme of no diabetic retinopathy (R0), background (R1), pre-proliferative (R2) and proliferative retinopathy (R3), | Regression | Age, BMI, duration of diabetes and known hypertension. |
| Manin 2014 | OSA | Ascertained (No participants had prior diagnosis of OSA or treatment with CPAP) | Diagnosed through polysomnography (AHI >= 10) | Retinopathy identified by retinal photography, ophthalmologic examination and/or previous laser therapy; | Regression | age, BMI, OSA (as a categorical variable), duration of diabetes, smoking (as a categorical variable), HbA1c and hypertension (as a categorical variable).\ |
| Banerjee 2013 | OSA | Not ascertained | Diagnosed through polysomnography (AHI >= 15) | NR | Regression | Age, gender and ethnicity, DM duration, insulin treatment and HbA1c, hypertension, and coronary artery disease. |
| Blegen 2021 | OSA | Ascertained (prior diagnosis with OSA based on testing documentation) | Diagnosed through polysomnographpolysomny (AHI >= 15) | funduscopic examination and medical-record review. | Regression | Factors significantly associated with cotton-wool spots (CWS) |
| Chen 2020 | OSA | Ascertained (No participants had prior diagnosis of OSA or treatment with CPAP) | Polysomnography: (AHI) >5 events/h (mild), 15–30 events/h (moderate) and AHI ≥30 (severe). | NR | Regression | NR |
| Banghoej 2016 | OSA | Ascertained (earlier diagnosis with OSA) | An AHI score ≥ 5/h is defined as OSA and OSA severity was defined according to the American Academy of Sleep Medicine (AASM) guidelines from 2009 (Epstein et al., 2009) as mild OSA (AHI ≥ 5 and b15/h), moderate OSA (AHI ≥ 15 and ≤30/h), and severe OSA (AHI N 30/h). | Digital fundus photography. | Regression | Gender, age, BMI, hypertension, diabetic nephropathy, autonomic neuropathy, and peripheral neuropathy |
| Altaf 2017 2017 | OSA | Ascertained (excluded known OSA at baseline) | using a portable multichannel device (Alice PDx; Philips Respironics, Best, the Netherlands. An apnea–hypopnea index (AHI) greater than or equal to 5 events per hour was consistent with an OSA diagnosis | Two-field 45-degree digital retinal images | Regression | ethnicity, age at diabetes diagnosis, diabetes duration, sex, glycated hemoglobin, BMI, systolic blood pressure, insulin use, number of antihypertensive agents, oral antihyperglycemic agents, and estimated glomerular filtration rate |
| Furukawa 2013 | OSA | Not ascertained | ODI >=5 | by the presence of haemorrhages, microaneurysms, soft and hard exudates, areas of neovascularisation or laser coagulation scars in at least one eye using florescence fundoscopy on dilated pupils within 3 months | Regression | sex, age, BMI, hypertension, hyperlipidaemia, current smoking, current drinking, stroke, ischaemic heart disease, duration of T2DM and HbA1c |
| Hermans 2014 | OSA | Not ascertained | Patients were formally classified into OSAS[] and OSAS[+] according to the current International Classification of Sleep Disorders Diagnostic and Coding Manual (ICSD-2), the diagnostic criteria of OSAS including: A, B, and D or C and D to satisfy the criteria: (A) at least one of the following: (i) patient complains of unintentional sleep episodes during wakefulness, daytime sleepiness, unrefreshing sleep, fatigue, or insomnia; (ii) patient awakens with breath holding, gasping, or choking; (iii) bedpartner reports loud snoring, breathing interruptions, or both during patient’s sleep. (B) Polysomnography shows: (i) 5 scoreable respiratory events (i.e., apneas, hypopneas, or respiratory effort-related arousals (RERAs)) per hour of sleep; (ii) evidence of respiratory effort during all or a portion of each respiratory event; or C. Polysomnographic recording showing: (i) 15 scoreable respiratory events (i.e., apneas, hypopneas or RERAs) per hour of sleep; (ii) evidence of respiratory effort during all or a portion of each respiratory event. (D) The disorder is not better explained by another sleep disorder; medical or neurologic disorder; medication use; substance abuse or alcohol | retinal examination by an experienced ophthalmologist and/or fluorescein angiography | None | - |
| Storgaard 2014 | OSA | Ascertained (Exclusion based on baseline OSA) | The frequency of obstructive events was reported as an apnoea–hypoponea index (AHI). The apnoea–hypoponea index is the number of apnoeas and hypopnoeas per hour of sleep. An apnoea–hypoponea index score ≥ 5/h was indicative of obstructive sleep apnoea | Diabetic retinopathy was classified as nil, simplex retinopathy and proliferative retinopathy based on digital fundus photography | Regression | DR not included |
| Laaban 2009 | OSA | Ascertained (prior diagnosis with OSA was excluded) | patients had mild SAS, as defined by a respiratory disturbance index (RDI) of 5–15; 19% had moderate SAS (RDI: 16–29) and 10% had severe SAS (RDI ≥ 30) | Patient's file (yes, no) | None | - |
| Adderley 2020 | OSA | Ascertained (excluded cases with existing outcome at baseline) | Record of a relevant diagnostic clinical code | Record of a relevant diagnostic clinical code | Matching | Age, sex, BMI, DM duration |

OSA: obstructive sleep apnea; DR: diabetic retinopathy; NR: not reported; AHI: apnea-hypopnea index; HTN: hypertension; DM: diabetes mellitus; HbA1C: glycated hemoglobin; BMI: body mass index; LCL-C: low-density lipoprotein-cholesterol; HDL-C: high-density lipoprotein-cholesterol; SBP: systolic blood pressure; DBP: diastolic blood pressure;
